# Effect of Temperature and Duration Time of Maceration on Nitrate Content of* Vernonia cinerea* (L.) Less.: Circumscribed Central Composite Design and Method Validation

**DOI:** 10.1155/2019/1281635

**Published:** 2019-05-02

**Authors:** Chaowalit Monton, Chitradee Luprasong

**Affiliations:** ^1^Drug and Herbal Product Research and Development Center, College of Pharmacy, Rangsit University, Pathum Thani 12000, Thailand; ^2^Sun Herb Thai Chinese Manufacturing, College of Pharmacy, Rangsit University, Pathum Thani 12000, Thailand

## Abstract

*Vernonia cinerea *(L.) Less. is a herbal plant in Family Asteraceae. It can be used as a smoking cessation aid due to the fact that it contains nitrate which can induce tongue numbness and cause less favor of cigarette smell and taste. The aim of this work was to investigate the effect of maceration temperature and time on the nitrate content of* V. cinerea. *A circumscribed central composite experimental design was applied in the work. Two factors (temperature and duration time) were investigated and two responses (yield of the extraction and nitrate content) were monitored. The high performance liquid chromatography using for quantitative analysis of nitrate content was validated. The HPLC response was linear (R^2^ = 1.000) in range of 10-100 *μ*g/mL. The HPLC method was specific, precise, and accurate. The maceration temperature and time were varied from 40 to 100°C and 10 to 60 min, respectively. Results showed that maceration at high temperature and long duration time gave the simultaneous high yield of the extraction and nitrate content. The prediction by the computer software, Design-Expert, was stable, reliable, and accurate. The optimal condition that provided simultaneous high yield of the extraction and nitrate content was achieved when extraction was at 99.5°C for 56.4 min.* V. cinerea* extracted using the optimal condition gave the yield of the extraction and nitrate content of 15.6% and 0.610%, respectively. In conclusion, maceration temperature and duration time had a positive effect on yield of the extraction and nitrate content of* V. cinerea. *Furthermore, the optimal condition in this work could be used as a guide for extraction of* V. cinerea *to obtain the high yield of the extraction as well as nitrate content.

## 1. Introduction

*Vernonia cinerea* (L.) Less. is a plant in Family Asteraceae. It has several activities such as antioxidant activity [1–5], anti-inflammatory activity [6, 7], and radioprotective activity [8]. Furthermore, it also has antitumor activity, antimicrobial activity, antidiabetic activity, etc. [9]. In Thailand, this plant is officially included in the National List of Essential Medicines use as a smoking cessation aid.* V. cinerea* contains a low content of nicotine, so it may act as nicotine replacement therapy. Naijitra and Cheoymang report nicotine in leaf extracts of* V. cinerea* is 0.115% [10]. In addition, Ketsuwan et al. report nicotine in flower extracts is 0.123% and in leaf extracts is 0.154% [3].* V. cinerea* also contains nitrate salt that can induce tongue numbness and cause less favor of cigarette smell and taste [11, 12]. Nitrate contents in* V. cinerea* stem extracts and leaf extracts are previously reported; 21% and 19%, respectively [3]. Among preparations using for smoking cessation,* V. cinerea* preparation have daily cost cheaper than that of standard preparations; bupropion, nicotine gums, or nicotine patches [13]. So,* V. cinerea *is an alternative aid of smoking cessation with cost-saving manner. There are a recent systematic review and meta-analysis of five randomized clinical trials with 347 smokers included; the results show that* V. cinerea* has potential efficacy for smoking cessation with no significant difference of adverse events between the treatment group and control group [14]. Nowadays,* V. cinerea* is developed into several dosage forms, i.e., tea [13, 15, 16], capsules [17], lozenges [18], juice [19], coffee [20], cookies [11], jelly candies [12], and pastilles [21].

The extraction of a plant is an important step to increase the concentration of the targeted plant bioactive compounds. Furthermore, it can provide a robust and reproducible extraction method that is independent of the variations of the plant raw materials [22]. The different conditions and different extraction techniques should be used for understanding the extraction selectivity of various plant active compounds [23]. The aim of this work was to investigate the effect of maceration temperature and time on the nitrate content of* V. cinerea. *The circumscribed central composite experimental design was applied in the work. Two factors (temperature and duration time) were investigated and two responses (yield of the extraction and nitrate content) were monitored. The method validation of high performance liquid chromatography (HPLC) for quantitative analysis of nitrate content was also performed. The authors expected that the optimal condition obtained from this work might be used as a guide for extraction of* V. cinerea *to obtain the simultaneous high of yield of the extraction as well as nitrate content.

## 2. Materials and Methods

### 2.1. Materials

Potassium nitrate and orthophosphoric acid (85%) were purchased from Carlo Erba Reagents, France. Octylamine was purchased from Sigma-Aldrich, USA. Methanol (HPLC grade) was purchased from Honeywell-Burdick & Jackson, USA.

### 2.2. Plant Raw Material

The whole plant of* V. cinerea *was harvested from Buachet District, Surin Province, Thailand, in August 2018. The plant was identified by Ajarn Nirun Vipunngeun, Department of Pharmacognosy, College of Pharmacy, Rangsit University. The voucher specimen was coded as CM-CC001-1-08-2018 and deposited at Drug and Herbal Product Research and Development Center, College of Pharmacy, Rangsit University. The fresh* V. cinerea* was cut into small pieces and sun-dried. Then, they were pulverized and stored in a dry place until use.

### 2.3. Extraction of Nitrate and the Optimization

The circumscribed central composite experimental design was applied in the work. Two factors were investigated: temperature and time. Temperature and time were varied from 40 to 100°C and 10 to 60 min, respectively. Traditionally, the plant was infused using boiling water and stand for a few minutes. In some cases, the decoction was used; the plant was boiled in hot water for approximately 15 min. So the selected temperature and time range of this work was based on the traditional use of this plant.* V. cinerea *powder was extracted by maceration technique. Water was used as an extraction solvent. The 50 mL of water was added to 5 g of* V. cinerea *powder in a 100-mL beaker. It was macerated in hot air oven (JS Research Inc., South Korea) in specific temperature and duration time as shown in Table 1, in which ten model conditions were designed. They were extracted for three times; the filtrates were pooled and then freeze-dried. The freeze-dried extracts were kept in a desiccator until use. The two responses were monitored: yield of the extraction and nitrate content. The optimization was performed using Design-Expert software (version 11.0.2.0) (Stat-Ease, Inc., USA). The 3D response surfaces of model conditions of yield of the extraction and nitrate content were produced. The predicted values vs. actual values plot and residuals vs. run plot were produced to ensure the reliability and stability of the prediction by the software. The desirability function was used to select the optimal condition [24, 25] that provided the simultaneous highest yield of the extraction and nitrate content.

### 2.4. HPLC Method Validation

The method validation was performed according to ICH guideline. Five topics were investigated: linearity and range, specificity, limit of detection (LOD) and limit of quantitation (LOQ), precision, and accuracy.

#### 2.4.1. Linearity and Range

A stock solution of potassium nitrate 16.3 mg (equivalent to nitrate 10 mg) was accurately weighed, dissolved in ultrapure water, and adjusted to the volume to 10 mL. It was diluted into five concentration levels including 100, 75, 50, 25, and 10 *μ*g/mL. They were filtered using 0.45 *μ*m pore size syringe filter. They were injected into the HPLC instrument (n=3) and a calibration curve of nitrate was constructed. Linear equation, coefficient of determination (R^2^), and test range were reported.

#### 2.4.2. Specificity

Specificity was determined by comparison of UV spectrum of the peak of standard nitrate with the peak of nitrate in* V. cinerea* extract. In case of the peak of* V. cinerea* extract, UV spectrum at three regions of the peak, upslope, center, and downslope, were monitored. The method was specific when UV spectrum at three regions of the peak of nitrate in* V. cinerea* extract was similar to UV spectrum of the peak of standard nitrate.

#### 2.4.3. LOD and LOQ

LOD and LOQ were calculated from the slope of the calibration curve and standard deviation (SD) of the response of a blank solution from ten injections. LOD and LOQ were calculated as (1) and (2):

(1) LOD=3.3σS

(2) LOQ=10σS

where *σ* was the SD of the response of the blank solution and S was the slope of the calibration curve.

#### 2.4.4. Precision

Repeatability and intermediate precision were determined. Three concentration levels of nitrate, 75, 50, and 25 *μ*g/mL, were prepared. They were analyzed by HPLC instrument in triplicate for each concentration. The percent relative standard deviation (%RSD) from the analysis of each day for three consecutive days was reported as repeatability. The %RSD of pooled results from three days was reported as intermediate precision.

#### 2.4.5. Accuracy

The accuracy was determined by the standard addition method and presented as percent recovery. Nitrate in three concentration levels, 75, 50, and 25 *μ*g/mL, was individually added to* V. cinerea* extract. They were analyzed by HPLC instrument in triplicate for each concentration and the percent recovery was calculated.

### 2.5. Quantitative Analysis of Nitrate Content

Nitrate content in* V. cinerea* was quantified using reversed-phase ion-interaction HPLC which was previously used for determination of nitrate in canned vegetable juices [26], vegetables [27], and sodium nitrate mouthwash [28, 29]. The quantitative analysis was performed by HPLC instrument Agilent 1260 infinity, Agilent Technologies, USA. The isocratic elution of 0.01 M octylammonium orthophosphate (pH 7.0) was used. It was prepared by dissolving 1.3 g octylamine in 30%v/v methanol, adjusting the pH with 10% orthophosphoric acid to 7.0, and finally adjusting the volume to 1,000 mL using 30%v/v methanol. The separation was done on ACE Generix column (150 mm × 4.6 mm, internal diameter, 5 *μ*m) with a temperature of 25°C. The flow rate of mobile phase was 0.8 mL/min. The injection volume was 10 *μ*L. The detection wavelength was 213 nm.

In case of* V. cinerea* extract, it was diluted by ultrapure water into the concentration of 2 mg/mL, filtered using 0.45 *μ*m pore size syringe filter, and injected into HPLC instrument. The nitrate content of* V. cinerea* extract was calculated from the calibration curve of nitrate. Mean and SD of nitrate content were reported.

## 3. Results and Discussion

### 3.1. Method Validation Results

**Figure 1:**
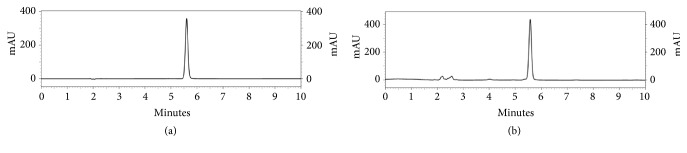
HPLC chromatograms of (a) nitrate (50 *μ*g/mL) and (b)* V. cinerea* extract extracted from condition 9 (2 mg/mL).

**Figure 2:**
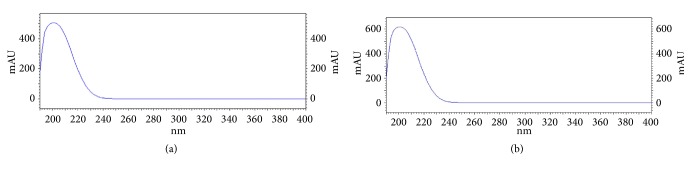
UV spectrums at (a) center of the peak of nitrate and (b) center of the peak of nitrate in* V. cinerea*.

The HPLC method had linear equation of y = 108,141x + 45,243 (R^2^ =1.000) in the test range of 10-100 *μ*g/mL. The LOD and LOQ were 10 and 40 ng/mL, respectively. The HPLC chromatograms of standard nitrate and* V. cinerea* extract are shown in Figure 1. Nitrate was eluted at the retention time of 5.6 min. According to the specificity of the analysis, this method was specific due to UV spectrum at upslope, center, and downslope of the peak of nitrate in* V. cinerea* extract was similar to UV spectrum of the peak of standard nitrate (Figure 2). Results of precision and accuracy are shown in Table 2. Repeatability of the analysis had %RSD less than 2% for the individual for three consecutive days. Moreover, intermediate precision performed from the analysis for three consecutive days was less than 5%. The accuracy represented as percent recovery had the value close to 100%; 97.58-102.46%. These results indicated that the HPLC method was precise and accurate.

### 3.2. Optimal Condition for Extraction of V. cinerea

**Figure 3:**
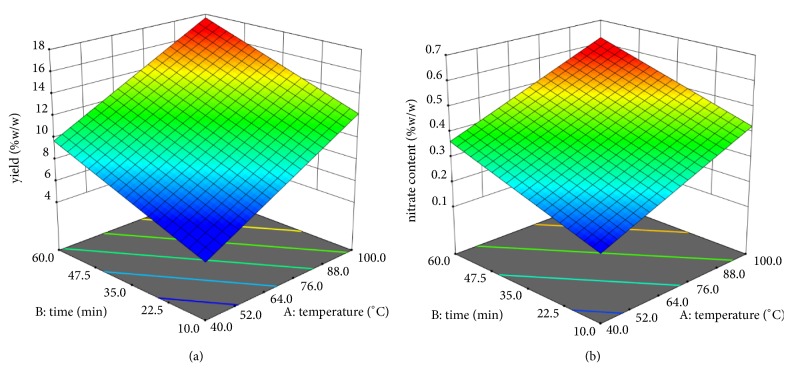
Response surfaces of model conditions of (a) yield of the extraction and (b) nitrate content.

Factors and responses of model conditions of yield of the extraction and nitrate content are shown in Table 1. Condition 6 was the best condition providing the highest yield of the extraction as well as nitrate content while Condition 5 provided the lowest yield of the extraction and nitrate content. The range of nitrate content in plant raw material was 0.175-0.578% dependent on the extraction condition. The nitrate content in extracts ranged from 2.9 to 4.1% (data not shown), which was lower than the previous report. Ketsuwan et al. reported that nitrate contents in* V. cinerea* stem extract and leave extract obtained from decoction were 21% and 19%, respectively, while they were not found in flowers extract [3].  Figure 3 shows the 3D response surfaces of model conditions of yield of the extraction and nitrate content. The results revealed that yield of the extraction and nitrate content was high when the maceration is done at high temperature and long duration time. Conversely, yield of the extraction and nitrate content were low when the maceration is done at low temperature and short duration time. The equations for prediction of yield of the extraction and nitrate content when macerate is at different temperatures and duration times are shown in (3) and (4), respectively.

(3) Yield  %=−1.868+0.131temperature+0.107time

(4) Nitrate  content  %=−0.049+0.004temperature+0.004time

The above 3D response surfaces and equations revealed that maceration temperature and duration time had a positive effect on the yield of the extraction and nitrate content of* V. cinerea*. Extraction temperature and extraction time are the important factors affecting the content of chemical constituents of several plants. When temperature increased, the content of phenolic compound, flavonoid, and condensed tannins of* Centella asiatica *increased. However, the extraction time had the optimum value; too long extraction time led to the decomposition of plant chemical compounds [30]. Lv et al. investigated the effect of extraction temperature and extraction time on the content of amygdalin of two plants, apricot-kernel and* Prunus tomentosa* using reflux extraction. They found that when temperature and duration time increased, the content of amygdalin increased. However, when the maximum content is reached, amygdalin content was gradually decreased due to its decomposition [31]. The longer extraction time might lead to thermal instability and degradation of thermolabile compounds. The long duration time could destroy some polysaccharides such as polysaccharides of* Hovenia dulcis* peduncles [32] and polysaccharides of* Angelica sinensis *[33]. Moreover, the high extraction temperature combined with long extraction time also led to degradation of polysaccharides of* Tricholoma mongolicum* [34], mulberry fruit [35], and* Astragalus cicer* [36]. The effect of temperature and time of pressurized hot water extraction on the polyphenolic content of* Thymus vulgaris* was evaluated. The results showed that the highest yield of some polyphenols such as hydroxycinnamic acids, flavones, flavonols/flavanones, and total polyphenols was obtained when extraction at 100°C for 5 min. Furthermore, they found that the higher extraction temperature and longer extraction time provided less diversity of above polyphenols. The 3,4-dihydroxyphenyllactic acid was the only phenolic compound that mostly extracted at high temperature while the nonphenolic antioxidant was favored at the higher extraction temperature and extraction time [37]. Dent et al. reported extraction temperature had a positive effect on mass fractions of rosmarinic acid and luteolin-3-glucuronide of* Salvia officinalis*, while extraction time had a positive effect only on mass fractions of luteolin-3-glucuronide [38]. Martins and da Conceicao revealed extraction time had a significant effect on rosmarinic acid and caffeic acid of* Apeiba tibourbou *extracted using ultrasound-assisted extraction while extraction temperature had no effect on both rosmarinic acid and caffeic acid [39]. Kuzmanović et al. extracted the phenolic compound from corn silage using high temperature and high pressure reactor. They varied several extraction parameters. The optimization results showed that temperature was the most significant factor affecting phenolic compound content [40]. The above results indicated that optimal temperature and time of the extraction procedure should be optimized to obtain the maximum content of targeted plant active constituents.

**Figure 4:**
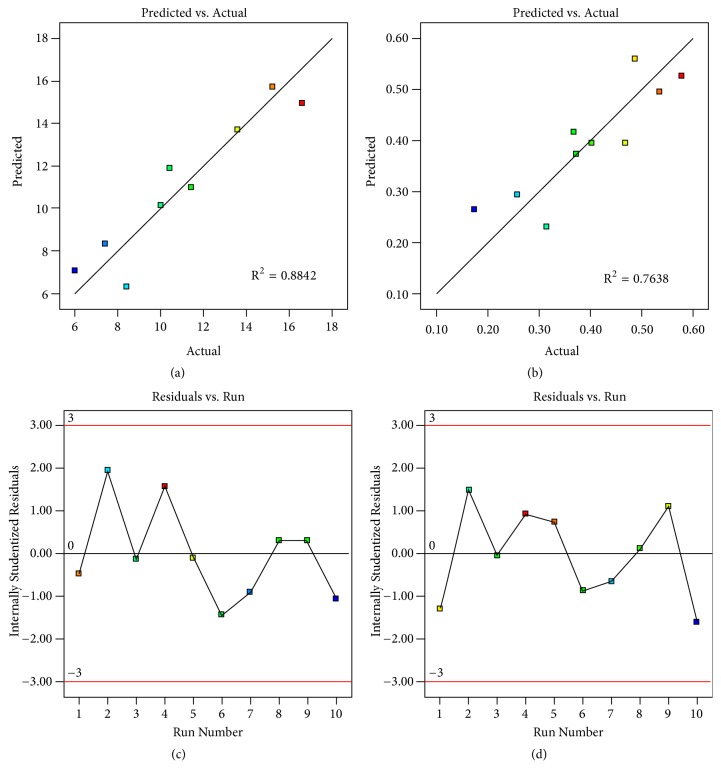
Predicted vs. actual value plots of model conditions of (a) yield of the extraction and (b) nitrate content and residuals vs. run plots of model conditions of (c) yield of the extraction and (d) nitrate content.

Figure 4 shows predicted vs. actual value plots and internally studentized residuals vs. run number plots of model conditions of yield of the extraction and nitrate content. The predicted vs. actual value plots had moderate to high R^2^ indicating that the predicted value was correlated to the actual (or experimental) value. The internally studentized residuals vs. run number plots displayed that the data was distributed within 95% confident interval. The above data could approve the reliability and stability of the prediction [41, 42]. The optimal condition provided the highest yield of the extraction and nitrate content obtained from computer software, Design Expert, were 99.5°C and 56.4 min. The desirability of the prediction was 1.000. According to the prediction value, extraction using this optimal condition gave the yield of the extraction and nitrate content of 17.2% and 0.610%, respectively while the experiments revealed that extraction using this optimal condition provided the yield of the extraction and nitrate content of 15.5% and 0.610%, respectively. Comparison between the predicted value and experimental value found that percent error of the prediction was -10.97% and 0% for yield of the extraction and nitrate content, respectively (Table 3). These results indicated that computer software could predict in an accurate manner. However, we suggested that extraction at 100°C could be used due to the fact that it was an easier manner for the extraction setting.

## 4. Conclusions

The HPLC was validated in this work and used for quantitative analysis of nitrate content of* V. cinerea*. The HPLC method had the linear response and it was specific, precise, and accurate. The circumscribed central composite experimental design was applied in the work. Effect of maceration temperature and duration time on yield of the extraction and nitrate content were investigated. The maceration at high temperature and long duration time provided the high yield of the extraction as well as nitrate content. This result indicated that the maceration temperature and duration time had a positive effect on yield of the extraction and nitrate content of* V. cinerea. *However, the other factors also affected the yield of the extraction and nitrate content of* V. cinerea *such as plant variation, harvesting location, harvesting period, extraction technique, and solid-to-solvent ratio. We suggested that the optimal condition reported in this work could be used as a guide for extraction of* V. cinerea *to obtain the simultaneous high yield of the extraction and nitrate content.

## Figures and Tables

**Table 1:** Factors and responses of model conditions of circumscribed central composite experimental design.

Condition	Factors				Responses	
	Temperature		Time		Yield of the extraction (%)	Nitrate content (%)
	Coded	Actual (°C)	Coded	Actual (min)		
1	-1	48.8	-1	17.3	8.4	0.315±0.008
2	1	91.2	-1	17.3	10.4	0.368±0.000
3	-1	48.8	1	52.7	10.0	0.372±0.000
4	1	91.2	1	52.7	15.2	0.486±0.000
5	-√2	40.0	0	35.0	6.0	0.175±0.000
6	√2	100.0	0	35.0	16.6	0.578±0.000
7	0	70.0	-√2	10.0	7.4	0.258±0.001
8	0	70.0	√2	60.0	13.6	0.536±0.001
9	0	70.0	0	35.0	11.4	0.467±0.000
10	0	70.0	0	35.0	11.4	0.402±0.000

**Table 2:** Precision and accuracy results.

Concentration (*μ*g/mL)	Precision (%RSD)				Spike concentration (*μ*g/mL)	Accuracy
	Repeatability			Intermediate		%Recovery
	Day 1	Day 2	Day 3			
25	0.24	0.03	0.09	0.50	25	102.46
50	0.09	0.04	1.12	0.67	50	97.58
75	0.07	0.07	0.13	0.28	75	101.68

**Table 3:** Predicted values, experimental values, and percent error of the prediction.

Responses	Predicted value	Experimental value	Error (%)*∗*
Yield of the extraction (%)	17.2	15.5±0.14	-10.97
Nitrate content (%)	0.610	0.610±0.001	0.00

*∗* %Error = (experimental value – predicted value)/experimental value × 100.

## Data Availability

The data used to support the findings of this study are available from the corresponding author upon request.
